# An Exploratory Survey-Based Evaluation of Primary Care Providers’ Knowledge and Diagnosis of Neglected Tropical Diseases in Texas

**DOI:** 10.7759/cureus.83262

**Published:** 2025-04-30

**Authors:** Daphne E Sanchez, Jay Wang, Gisela M Ortega, Han Huynh, Karimeh Ortiz, Rebecca L Sanchez

**Affiliations:** 1 Department of Applied Biomedical Sciences, University of the Incarnate Word School of Osteopathic Medicine, San Antonio, USA

**Keywords:** disease reporting, neglected tropical disease, primary care providers, texas, vector-borne

## Abstract

Objective: The purpose of this survey is to determine the current level of neglected tropical disease (NTD) knowledge among primary care providers (PCPs), determine the ability of Texas PCPs to identify and diagnose a patient with an NTD, and assess the current knowledge of and identify barriers to NTD reporting mandates and procedures by Texas PCPs.

Background: As global temperatures increase, Texas' geographic outlook continues to change, causing vectors of NTDs to widen their endemic zones. Furthermore, PCPs can range from nurse practitioners to physicians, with varying representations depending on location and availability. As such, it is pertinent to understand the current knowledge gaps of NTDs, their clinical presentation, and the proper diagnostic tools.

Methods: From February 2024 to July 2024, a 23-question Qualtrics survey (Qualtrics, Provo, UT) was distributed via external and social media outreach, targeting primary health providers, including physicians (Doctor of Medicine/Doctor of Osteopathic Medicine, DO), physician assistants, and nurse practitioners, in any Texas healthcare setting. Questions inquired about knowledge regarding NTDs, appropriate diagnostic tests, and knowledge and attitudes toward reporting the disease in the state of Texas.

Results: A total of 21 survey responses were obtained, with nine being excluded due to incompletion. The majority of survey respondents were DOs (58.3%), practiced family medicine (41.7%), and practiced in Bexar County (58.3%), with an estimated average of 11 years of practice experience. As defined by the World Health Organization, 58.3% of respondents reported never having been diagnosed with an NTD. Seventy-five percent of respondents were aware of the proper reporting requirements outlined in Texas Administrative Code Chapter 97, Title 25. However, 50% needed to be made aware of the reportable disease list and the required timeline for reporting; 66.7% of respondents cited knowledge of the disease as the main barrier to incomplete reporting.

Discussion: This study demonstrates that only a few providers in Texas, namely Bexar County, could accurately identify NTD diagnostic tests, which may highlight the lack of knowledge of NTD evaluation. Due to the nature of vector-transmitted NTDs, it is imperative to explore widespread educational methods for providers. Expanding access to online reporting platforms for outpatient providers is essential to mitigate barriers and streamline the process. This study was severely limited by selection bias, small sample size, generalizability, and geographic representation, and thus serves as a preliminary study to inform future research.

## Introduction

Neglected tropical diseases (NTDs) encompass a wide range of conditions that significantly impact the health and economic stability of over one billion individuals worldwide [1]. Labeled as "neglected," these diseases are primarily found in developing countries and tropical regions within Africa, Asia, and Latin America and have been historically under-addressed by global public health initiatives [2]. NTDs inflict the most significant toll on impoverished communities, particularly women and children in endemic areas. However, there is rising concern about the disease burden among the poor in wealthy, nonendemic countries [3,4]. In the United States (U.S.), NTDs primarily affect people of color in racially segregated urban areas, the U.S.-Mexico borderlands, and people of all races living in impoverished rural areas like the Mississippi Delta and Appalachia [4].

Understanding the epidemiology of NTDs is complex and often intertwined with environmental factors, especially those that support vector populations [4]. Many NTDs are vector-borne, involving arthropods such as mosquitoes and triatomine bugs, and are maintained through animal reservoirs. They exhibit intricate life cycles and are not often screened for in patients. Three NTDs defined by the World Health Organization (WHO) will be discussed in this paper: Chagas disease, dengue fever, and chikungunya. Additionally, the scope of this paper will be expanded to consider other related arboviral infections with high disease burden, including West Nile virus and Zika virus [2].

Climate change

Climate change influences global climates, increasing temperatures and altering precipitation patterns. Increased temperatures correlated with higher mosquito breeding, survival, and biting rates, which can be seen with both the Culex species that transmit West Nile virus and the Aedes species that transmit dengue fever, chikungunya, and Zika virus [5]. One study found that Culex mosquito development sped up in temperatures above 16°C, increasing their numbers and transmission to birds (the primary reservoir) and humans [6]. Warmer than average temperatures were seen in the 2012 West Nile virus outbreak in Texas, which resulted in nearly 1,900 cases and 89 deaths [5]. In addition, Aedes mosquitoes increase their reproduction and feeding activity dramatically in temperatures above 26°C [7]. Local transmission of chikungunya and Zika was reported in the Texas counties of Cameron and Hidalgo in 2015 and 2016, respectively [5]. These local cases and the documented presence of Aedes mosquitoes locally indicate that changes in Texas weather support the spread of virus-infected Aedes mosquitoes northward from Mexico to Texas [5,8]. As dengue fever incidence also positively correlates with local temperature, precipitation, and humidity, it can be inferred that the virus will follow a similar northward travel as its counterparts [9]. Furthermore, triatomine bugs that transmit *Trypanosoma cruzi*, the causative agent of Chagas disease, have also been found in Texas [10]. While the triatomines found in Texas rarely colonize homes like those in Central and South America, over 50% of the bugs found and tested in Texas have been positive for *T. cruzi* [10,11]. Tracking triatomine populations has also found increased geographic range and activity in Texas, with climate change and increased temperatures [11]. Consequently, climate change may raise average temperatures, impacting vector breeding, shortening the incubation period, and increasing the vectors' ability to transmit arboviral diseases [9].

Urbanization and population growth

From 1997 to 2017, Texas' population increased by 10 million, averaging 470,000 annually [12]. This population growth has led to the fragmentation of agricultural and rural areas, with a significant decrease in working lands in the Texas borderlands since 1997 [13]. Rapid urbanization in cities like San Antonio and Austin will double their populations by 2040 [14]. This urban expansion reduces wildlife habitats, potentially increasing sylvatic transmission spillover of NTDs as vectors adapt to living in wildlife areas near human residences [5]. Studies have shown that hunting and camping can increase the risk of sylvatic transmission, as demonstrated by infected wood rats, opossums, and armadillos in Texas [5,15].

Surveillance and reporting

Many mosquito-related diseases, including chikungunya, dengue fever, West Nile virus, and Zika virus, lack commercially available human vaccines or therapies, making mosquito surveillance and control crucial [16]. The Texas Department of State Health Services' (DSHS) Arbovirus Surveillance Program aims to detect arboviruses in mosquitoes and *T. cruzi *in triatomine bugs before human disease development. However, budget cuts over the last decade have limited their capacity, leading to this testing being outsourced or limited to only bugs implicated in human exposure [17,18]. The Western Gulf Center of Excellence for Vector-Borne Diseases (VBD) highlights the U.S.'s unpreparedness for widescale VBD due to an incomplete understanding of VBD emergence, diminished surveillance capacity, and increased vector control challenges [19].

Dengue fever cases are reported to the Centers for Disease Control and Prevention through ArboNET, but surveillance for other VBDs remains limited. The lack of monitoring and underreporting of cases due to mild or asymptomatic presentations and limited testing sites exacerbate this issue [20,21]. Recommendations include educating healthcare providers early in their graduate education on VBDs, emphasizing epidemiology, clinical spectrum, diagnostics, management, and reporting responsibilities. The One Health initiative suggests a collaborative approach involving public health, agricultural health, veterinary health, and environmental health sectors to address biosecurity concerns [21,22].

Prevention methods

Proposed prevention methods for NTDs involve multifactorial approaches targeting vector transmission, such as spraying mosquito breeding grounds with larvicide and using adulticide fumigation in households [23]. While vector breeding ground spraying is already a common practice in some Texas cities, it is unknown how individual Texas residents will react to the use of household fumigation, and benefits and risks, as well as individual attitudes, must be weighed [24]. Public health interventions by the WHO include innovative disease management, large-scale preventative treatment, integrated vector management, and access to water, sanitation, and hygiene. Raising awareness among healthcare professionals and enhancing their ability to screen for NTDs can promote favorable outcomes in prevention [23].

To aid in the prevention and mitigation of vector-transmitted NTDs in Texas, this study aims to 1) determine the current level of NTD knowledge among primary care providers (PCPs), 2) assess the ability of Texas PCPs to identify and diagnose a patient with an NTD, and 3) assess the current knowledge of and identify barriers to NTD reporting mandates and procedures by Texas PCPs.

## Materials and methods

This descriptive cross-sectional study was conducted from February 2024 to July 2024. Individuals included in the study were healthcare providers practicing in Texas with one of the following degrees: Doctor of Medicine (MD), Doctor of Osteopathic Medicine (DO), Master of Physician Assistant Studies (MPAS), Doctor of Nursing Practice (DNP), or Master of Science in Nursing (MSN). No restrictions were placed on the type of healthcare setting or specialty, although participant recruitment prioritized primary care specialties. Exclusion criteria included providers not actively practicing, individuals under 18, and healthcare providers who do not hold the aforementioned degrees, including but not restricted to doctors of physical therapy, doctorates of occupational therapy, doctors of chiropractic, registered nurses (Bachelor of Science in Nursing and Associate of Science in Nursing), medical assistants, technicians, and other supportive healthcare staff. Primary healthcare providers were contacted, and data were collected via convenience and snowball sampling. Additional potential participants were recruited through Texas professional organizations, including the Bexar County Medical Society, the Texas Academy of Physician Assistants, and the American Association of Nurse Practitioners. Healthcare providers were also directly messaged through social media platforms, such as Instagram, Twitter, and LinkedIn. Recruitment efforts were extensive, and survey links and requests for participation were distributed to over 200 healthcare providers. However, due to snowball sampling methods, the exact number is unknown.

The questionnaire was administered through an anonymous Qualtrics survey link (Qualtrics, Provo, UT). The survey consisted of 23 items divided into three parts (see the Appendix). The first part collected demographic data that included the terminal degree of the providers (MD/DO/MPAS/DNP/MSN), healthcare specialty (e.g., family medicine, internal medicine, and emergency medicine), average patient load, and descriptions of the provider's medical practice (e.g., rural/urban, average patient load, and county location of practice). The second part of the survey addressed provider exposure to NTDs, including previous patient encounters, their diagnostic testing knowledge, and clinical presentations that would make them suspect NTD diagnoses. The last part of the survey asked providers about their knowledge and attitudes toward reporting reportable illnesses to public health authorities. The survey tool was validated via expert review by doctoral faculty in the Department of Biomedical Sciences at the University of the Incarnate Word School of Osteopathic Medicine. Other medical education faculty excluded from study participation performed pilot testing of the survey and provided feedback when necessary.

The University of the Incarnate Word institutional review board reviewed and approved the study protocol (no. 2024-1515-NRR-v3.6607). It was determined that the study did not meet the federal regulatory requirements for human subjects research under 45 CFR 46.102(d) and was categorized as not human subjects research. The confidentiality of the study participants' identities was maintained throughout the study by keeping their information confidential. This was a voluntary and noncompensated survey, and all participants were asked to provide their consent via the Qualtrics link before completing the remaining survey responses.

Statistical analysis

Anticipated methods of statistical analysis included analysis of variance (ANOVA) and chi-square tests. ANOVA interaction tests were to be used to determine any interactions between NTD diagnostic and reporting knowledge and NTD diagnosis and reporting rates, as well as any interactions between provider characteristics (e.g., specialty, location, and type of practice) and NTD diagnosis and reporting rates. Following any ANOVA analysis demonstrating a significant difference (p < 0.05), a post hoc Tukey test would have been used to identify which variables in the ANOVA were significantly different. Chi-square tests were used to determine any interactions between provider characteristics (e.g., rural vs. urban) and NTD diagnostic and reporting knowledge. The calculated number of subjects necessary to achieve a power of 0.8 and a significance level of 0.05 with the anticipated number of comparison categories was 110. Statistical analyses were not conducted to avoid random error associated with the small sample size and subsequent low power and low significance. Instead, descriptive statistics were used, and the data were presented in tables with frequencies and percentages of respondents' answer choices.

## Results

Characteristics of survey respondents

A total of 21 participants participated in the study, but nine were excluded from data analysis due to incomplete survey responses. The basic characteristics of the studied population are shown in Table 1. The majority of survey respondents were DOs (58.3%), practiced family medicine (41.7%), and practiced in Bexar County (58.3%). Practice settings varied, including private practice, community centers, and urgent care centers, with an estimated average of 11 years of practice experience.

**Table 1:** Demographics and general information of survey respondents (n = 12) Responses to Questions 1-5 of the survey have been reflected MD: Doctor of Medicine; DO: Doctor of Osteopathic Medicine; MPAS: Master of Physician Assistant Studies; MSN: Master of Science in Nursing

Demographics	n (%)
Educational degree	
DO	7 (58.3)
MD	3 (25)
MPAS	1 (8.3)
MSN	1 (8.3)
Practice specialty	
Emergency medicine	1 (8.3)
Family medicine	5 (41.7)
Geriatrics	1 (8.3)
Internal medicine	3 (25)
Practice facility type	
Private practice	4 (33.3)
Community center	2 (16.7)
Hospital	4 (33.3)
Hospital: urgent care	1 (8.3)
All	1 (8.3)
County of practice	
Bexar	7 (58.3)
Gillespie	1 (8.3)
Harris	1 (8.3)
Tarrant	2 (16.7)
Travis	1 (8.3)
Number of years practicing in healthcare	
0-1	1 (8.3)
2-3	4 (33.3)
4-5	1 (8.3)
16-17	2 (16.7)
20 or more	4 (33.3)

Knowledge and practices regarding NTDs

Provider experiences, practices, and knowledge of NTDs are shown in Tables 2, 3. Regarding the diagnosis and reporting of NTDs, 58.3% of respondents had never diagnosed an NTD as defined by the WHO. Among those who had been diagnosed with NTDs, scabies and other ectoparasites (38.5%) and Chagas diseases (15.4%) were the most common. For providers who had not diagnosed NTDs, 85.7% indicated they would report prospective patient encounters to the state health department, while 14.3% would contact their local health department.

**Table 2:** NTD knowledge (n = 12) Responses to questions 9, 9a1, 9b, and 10 of the survey have been reflected NTD: neglected tropical disease

Survey question and response	n (%)
During your time in practice, how many NTDs (defined by the World Health Organization as a diverse group of conditions caused by a variety of pathogens and associated with devastating health, social, and economic consequences) have you diagnosed?	
0	7 (58.3)
2	2 (16.7)
3	1 (8.3)
4	1 (8.3)
20	1 (8.3)
If you needed to report an NTD case, who would you contact?	
Local health department	1 (14.3)
State health department	6 (85.7)
Which of the following diseases have you diagnosed?	
Chagas (American trypanosomiasis)	2 (15.4)
Dengue fever	1 (7.7)
Leishmaniasis	1 (7.7)
Scabies and other ectoparasites	5 (38.5)
Snakebite envenoming	1 (7.7)
Soil-transmitted helminthiases (including the roundworm, *Ascaris lumbricoides*; the whipworm, *Trichuris trichiura* and hookworms, *Necator americanus* and *Ancylostoma duodenale*)	1 (7.7)
Taeniasis/cysticercosis (via *Taenia solium*, *Taenia saginata*, and *Taenia asiatica*)	1 (7.7)
Zika virus	1 (7.7)
What symptom(s) is/are most indicative of an NTD based on your experience? (Select all that apply)	
Fever	11 (27.5)
Diarrhea	4 (10)
Vomiting	1 (2.5)
Body aches	5 (12.5)
Rash	6 (15)
Bleeding	1 (2.5)
Headaches	1 (2.5)
Neck stiffness	3 (7.5)
Pain behind the eyes	1 (2.5)
Excessive sleepiness	3 (7.5)
Seizures	3 (7.5)
Loss of consciousness	1 (2.5)

**Table 3:** NTD diagnostic test knowledge (n = 12) Responses to question 11 of the survey have been reflected NTD: neglected tropical disease; ELISA: enzyme-linked immunosorbent assay; IFA: indirect fluorescent antibody assay; RT-PCR: reverse transcriptase-polymerase chain reaction; PCR: polymerase chain reaction

In your opinion, which diagnostic testing is most appropriate for the following NTDs?	n (%)
Chagas	
Clinical approach	2 (16.7)
Microscopy	3 (25)
ELISA	1 (8.3)
IFA	1 (8.3)
RT-PCR	1 (8.3)
Combination: microscopy + PCR	4 (33.3)
Combination: ELISA/IFA + PCR	-
Chikungunya	
Clinical approach	2 (16.7)
Microscopy	-
ELISA	2 (16.7)
IFA	1 (8.3)
RT-PCR	3 (25)
Combination: microscopy + PCR	1 (8.3)
Combination: ELISA/IFA + PCR	3 (25)
Dengue fever	
Clinical approach	4 (33.3)
Microscopy	-
ELISA	2 (16.7)
IFA	-
RT-PCR	2 (16.7)
Combination: microscopy + PCR	1 (8.3)
Combination: ELISA/IFA + PCR	1 (8.3)
West Nile virus	
Clinical approach	2 (16.7)
Microscopy	-
ELISA	3 (25)
IFA	1 (8.3)
RT-PCR	3 (25)
Combination: microscopy + PCR	-
Combination: ELISA/IFA + PCR	3 (25)
Zika virus	
Clinical approach	3 (25)
Microscopy	-
ELISA	2 (16.7)
IFA	1 (8.3)
RT-PCR	3 (25)
Combination: microscopy + PCR	-
Combination: ELISA/IFA + PCR	3 (25)

A list of symptoms (Table 2) was presented to providers to determine which clinical presentations were most suspicious of NTDs. The top three symptoms were fever (27.5%), rash (15%), and body aches (12.5%). These were followed by diarrhea (10%), neck stiffness (7.5%), excessive sleepiness (7.5%), and seizures (7.5%).

Providers had varied responses when questioned on appropriate diagnostic tests for Chagas, chikungunya, dengue fever, West Nile, and Zika, as represented in Table 3. For Chagas, 33.3% of respondents would order combination enzyme-linked immunosorbent assay (ELISA)/indirect fluorescent antibody assay (IFA) and polymerase chain reaction (PCR) tests. For Chikungunya, 25% of participants chose reverse transcriptase PCR (RT-PCR) and a combination of ELISA/IFA and PCR tests. Diagnostics for dengue fever were based on clinical presentation (33.3%). For West Nile, responses were split evenly among microscopy, RT-PCR, and a combination of ELISA/IFA and PCR (25% each). For Zika, providers were equally divided among clinical presentation, RT-PCR, and a combination of ELISA/IFA and PCR (33.3% each). Plaque reduction neutralization was offered as a diagnostic option for the listed NTDs, but none of the providers chose this as a preferred diagnostic.

Knowledge and attitudes regarding reportable diseases

Respondent knowledge and attitudes regarding reportable diseases are summarized in Table 4, where 75% of respondents knew of the Texas Administrative Code Chapter 97, Title 25, which states that "[h]ealth care providers, hospitals, laboratories, schools, and others are required to report to Texas DSHS patients who are suspected of having a notifiable condition." However, 50% needed to be made aware of the reportable disease list and the required timeline for reporting. Only one respondent received communication from the Texas Department of Health regarding changes in Texas' reportable disease list. Concerning the timing and responsibility for reporting, 58.3% believed reporting should occur when the disease is laboratory-confirmed. Others believed it depended on suspicion of diagnosis (16.7%) or when the patient met clinical criteria (25%). Most respondents (41.7%) felt that healthcare providers were best suited to report, followed by laboratory managers/supervisors (25%), infection control personnel (16.7%), and automated methods via electronic medical records (8.3%).

**Table 4:** Knowledge and attitudes of reporting diseases (n = 12) Responses to questions 13-23 of the survey have been reflected

Survey question and response	n (%)	
Before taking this survey, were you aware of Texas law that states that "[h]ealth care providers, hospitals, laboratories, schools, and others are required to report to Texas Department of State Health Services (DSHS) patients who are suspected of having a notifiable condition?"		
Yes	9 (75)	
No	3 (25)	
Before taking this survey, were you aware of the reportable disease list for contacting public health authorities about a reportable disease set forth by the Department of Health Commissioner?		
Yes	6 (50)	
No	6 (50)	
Before taking this survey, were you aware of the required timeline for contacting public health authorities about a reportable disease set forth by the Department of Health Commissioner?		
Yes	6 (50)	
No	6 (50)	
Do you receive communication from the Texas Department of Health (either electronic or standard mail) regarding changes in Texas’ reportable disease list?		
Yes	1 (8.3)	
No	11 (91.6)	
In your opinion, when is the best time to contact public health authorities regarding a patient with a reportable disease?		
Upon suspicion of the diagnosis	2 (16.7)	
When the disease is laboratory-confirmed	7 (58.3)	
When the patient clearly meets clinical criteria	3 (25)	
Deficits in which of the following knowledge areas may most contribute to INCOMPLETE reporting?		
Mechanism for submitting a report to public health	2 (16.7)	
Proper suspicion of the diagnosis	1 (8.3)	
Requirement that providers report	1 (8.3)	
Which diseases are reportable	8 (66.7)	
Deficits in which of the following knowledge areas may most contribute to DELAYS in reporting?		
Mechanism for submitting a report to public health	2 (16.7)	
Proper suspicion of the diagnosis	3 (25)	
Requirement that providers report	4 (33.3)	
Which diseases are reportable	3 (25)	
In your opinion, who is best suited to contact public health authorities about a patient with a reportable disease?		
Automated (via electronic medical record)	1 (8.3)	
Healthcare provider	5 (41.7)	
Infection control personnel	2 (16.7)	
Laboratory manager/supervisor	3 (25)	
Other	-	
Any of the above	1 (8.3)	
What method of reporting is most convenient for you?		
Secure email	2 (16.7)	
Secure web-based form	10 (83.3)	
Reportable diseases have variable time frames for reporting. If you had a reportable disease to submit, how would you determine the required time frame to report?		
Access the local or state department of health website	5 (41.7)	
Call the local or state health department	2 (16.7)	
Discuss with the hospital infection control	2 (16.7)	
Review posted reportable disease requirements	3 (25)	
What is your primary concern regarding the notification of a reportable disease to public health authorities?		
Concerns about violating patient privacy	1 (8.3)	
Not worthwhile if the disease is not highly contagious/fatal	2 (16.7)	
Reporting procedures are burdensome	6 (50)	
Unsure which diseases are required to report	3 (25)	

Regarding barriers to reporting, 66.7% cited knowledge of the disease as the main barrier to incomplete reporting. Other barriers included the mechanism for reporting (16.7%), proper suspicion of diagnosis (8.3%), and the requirement for providers to report (8.3%). For delayed reporting, 33.3% pointed to incomplete knowledge of reporting requirements as the primary reason, followed by mechanisms for reporting (16.7%), proper suspicion of diagnosis (25%), and which diseases to report (25%).

When questioned about their preferred reporting methods, 83.3% of respondents favored secure web-based forms, while 16.7% preferred secure email formats. When questioned on how to determine time frames for reportable diseases, 41.7% would access local or state health department websites, 25% would review posted reportable disease requirements, and the remaining providers would either call local or state health departments (16.7%) or discuss with hospital infection control (16.7%).

Half of the respondents found that reporting procedures needed to be more manageable. Additionally, 25% were unsure which diseases required reporting, 16.7% believed that reporting nonhighly contagious/fatal diseases was not worthwhile, and 8.3% had concerns about violating patient privacy.

## Discussion

The preliminary results from this exploratory survey confirm our hypothesis that Texas healthcare providers have limited exposure to NTDs. Those with experience commonly encountered scabies and or have sparse patient encounters with vector-associated NTDs like Chagas disease or Zika, suggesting a potential gap in the capacity of Texas PCPs to respond to the projected incidence and prevalence of such diseases as climate change progresses. However, the demographics of survey respondents demonstrated representation mainly from Texas counties that contained major cities and urban populations. This may highlight that urban and suburban healthcare providers, rather than rural providers, have a knowledge gap. A follow-up study will focus on rural providers' knowledge and increase in rural provider participation in future studies.

These findings demonstrate a lack of NTD experience. This is particularly relevant in light of previous research on Chagas disease knowledge among U.S. pediatric cardiologists, which showed that 87% of the 119 sampled had never encountered a case. In addition, 72% had never tested for it, and 85% were unsure of their ability to recognize it. In addition, most did not consider it in their differential diagnosis unless the patient was a Latin American immigrant, lacking awareness of the bug and parasite presence in the southern U.S. [25]. Furthermore, a study focusing on the effectiveness of learning modules in educating providers about Chagas, dengue, and West Nile virus found that this knowledge was relevant for identifying, diagnosing, and treating NTDs in Latinx immigrant communities and U.S. travelers primarily. While this study recognized climate change as a factor in NTD spread, it emphasized travel-related cases rather than the northward spread of the disease and its vectors [26]. Interestingly, a study on obstetrician/gynecologist knowledge of the Zika virus revealed that after Zika was declared a public health emergency in 2016, provider knowledge increased regarding symptoms, virus characteristics, detection, treatment, and local transmission rates [27]. This indicates that as the incidence increases, providers may respond accordingly by addressing their own knowledge gaps, as advised by public health officials.

The results from this study have demonstrated that only a few providers could accurately identify the diagnostic process needed for the following vector-transmitted diseases: Chagas, chikungunya, dengue fever, West Nile virus, and Zika virus. Diagnostic confirmation of Chagas disease requires a history of living in a Latin American country, along with the detection of trypomastigotes on microscopy or PCR [28], which 33.3% of survey participants accurately selected. RT-PCR is used to diagnose chikungunya and Zika virus, and 25% of respondents demonstrated knowledge of this [29,30]. ELISA is the gold standard for West Nile virus, which 25% of providers chose [31]. Finally, the diagnosis of dengue fever depends on the clinical presentation [32]; 33.3% of respondents accurately displayed this knowledge. These findings indicate that respondents may benefit from a comprehensive review of these diseases, including updated guidance on current evidence-based practices.

Despite most providers being aware of their obligation to report diseases, there is concern that 50% need to be made aware of which diseases to report and how to report them. Additionally, results showed knowledge discrepancies in the timing of reports, ranging from waiting for clinical presentations to confirmation of diagnostic criteria. Additionally, 50% of respondents felt that reporting procedures are burdensome. Due to the nature of vector-transmitted NTDs, it is of great interest to public health to improve awareness of reporting timelines and reduce the inconvenience of the reporting process. In Texas, healthcare providers, laboratory officials, and local health department officials can report notifiable diseases to the Texas DSHS via an online web portal through the Texas National Electronic Disease Surveillance System (NEDSS). This system enables the submission of electronic laboratory reports for hospitals and laboratories that are enrolled and onboarded in accordance with Texas DSHS requirements. However, it is not currently accessible to outpatient healthcare providers unless they share an electronic health record system with an enrolled hospital. Outpatient providers can alternatively use Electronic Case Reporting via an Association of Public Health Laboratories Informatics Messaging Service (AIMS) platform, but they must be enrolled in this service. This service incurs costs for the enrollee, which may be burdensome for some outpatient providers [33]. For providers not integrated with NEDSS or other electronic systems, the Texas DSHS provides downloadable reporting forms, which can be submitted via secure fax to local health departments. Additionally, the Texas DSHS provides website and contact information for the appropriate local health department office, allowing healthcare providers to report by phone with 24/7 access [33,34]. Most respondents confirmed they preferred online reporting methods (83.3%). Improving accessibility for outpatient clinics to electronic case reporting services could help simplify the reporting process for many providers and improve timely compliance with mandated reporting requirements.

Although many found the protocols burdensome, most respondents acknowledged it was within their scope and responsibilities to report. Standardized procedures and more frequent dissemination of updated reportable disease lists could address these issues and improve reporting compliance. Examples of addressing this are to work with professional healthcare organizations, such as county medical societies and state organizations (e.g., Texas Osteopathic Medical Association or Texas Chapter of the American College of Physicians). Similar to the study by Wrench et al. [26], these partnerships can emphasize the use of online learning modules or interactive case-based workshops while offering incentives for continuing medical education (CME) credits. Additionally, electronic medical record systems could integrate alerts with information on reporting responsibilities and provide links to relevant portals.

It is important to note that this study has limitations, including a small sample size despite extensive efforts to contact potential participants. As a result, inferential statistical analyses were not possible, and any results were not generalizable. The sample was also geographically skewed, with a majority of respondents practicing in Bexar County. This urban-centric representation limits insight into the experiences of rural and small-town providers. This is an important consideration given that rural areas and smaller towns face different risks, especially those with closer proximity to Mexico, an endemic area for these NTDs. Additionally, the recruitment method using convenience and snowball sampling likely introduced selection bias, attracting providers already engaged with professional networks or interested in infectious diseases or CME efforts, which may not reflect the broader population of PCPs. The self-reported nature of the survey data may also introduce information bias and recall bias, particularly in areas assessing knowledge, practices, or attitudes. For example, respondents may have overestimated their familiarity with diagnostic protocols or underreported uncertainty in recognizing NTD symptoms. Furthermore, while the survey aimed to evaluate diagnostic knowledge, it did not account for variations in clinical resources across different practice settings, which could significantly affect a provider's ability to implement diagnostic strategies, especially in underresourced or rural clinics.

Despite these limitations, the study's exploratory nature has identified prospective avenues and gaps for further research. For instance, a more concentrated distinction between rural and urban healthcare providers could provide valuable insights. Additionally, as most respondents found reporting protocols burdensome and expressed a preference for online reporting portals, future research should explore any potential technological barriers in different healthcare settings (e.g., federally qualified health centers vs. private practices). In the meantime, this study provides clear evidence that Texas PCPs must prepare to address the growing concerns of vector-transmitted NTDs secondary to increased global temperature. The limited experience and exposure to NTDs and systemic gaps in the reporting system are vital factors that underscore the issue's urgency. Immediate action is needed to address these challenges.

## Conclusions

Due to the ongoing concerns about vector-transmitted NTDs and global warming, it is pertinent for Texas infrastructure to establish and preemptively begin planning methods to mitigate disease spread. This exploratory study highlights key gaps in provider familiarity with NTDs and inconsistencies in knowledge and practices related to disease reporting. The results underscore the need for coordinated, statewide training initiatives that provide targeted and up-to-date knowledge of NTD diagnosis and the timelines for public health reporting requirements. Integrating these training efforts into CME programs, professional conferences, and electronic medical record systems could enhance clinical readiness and improve compliance with disease surveillance protocols. This study additionally found that providers desire a streamlined method to satisfy reporting requirements, and addressing technological barriers should be a goal for future public health efforts, particularly in outpatient and underresourced settings. To ensure these interventions are impactful and inclusive, future studies must address the limitations of this exploratory work. Expanding the sample size, including providers from rural and underrepresented areas, especially those near the U.S.-Mexico border, and performing inferential statistical analysis would improve generalizability and illuminate regional disparities. This study provides a foundation for future research and education efforts by identifying knowledge gaps and operational barriers. Addressing these challenges through systematic, scalable training and reporting improvements will be essential in building a responsive and informed PCP workforce capable of adapting to the evolving landscape of NTD risk in Texas.

## Appendices

Screening/qualifying questions

1. What degree(s) do you hold? (Select all that apply)

DNP

MSN

MD

DO

MPH

MPAS

Other terminal degree (please specify): ______

2. What is your specialty and/or subspecialty? (Select all that apply)

Family medicine

Internal medicine

Pediatrics

Geriatrics

Obstetrics/Gynecology (OB/GYN)

Emergency medicine

Infectious disease

Hospital medicine

Other (please specify): ______

3. What kind of facility or facilities do you work?

Private practice

Community-based center

Urgent care

Hospital

Other (please specify): ______

4. In what county in Texas do you practice?

Anderson

Angelina

Bastrop

Baylor

Bee

Bell

Bexar

Bowie

Brazoria

Brazos

Brown

Burnet

Cameron

Cherokee

Childress

Collin

Colorado

Cooke

Dallas

Dawson

De Witt

Deaf Smith

Denton

Eastland

Ector

El Paso

Ellis

Falls

Fort Bend

Freestone

Gillespie

Gonzales

Gray

Grayson

Gregg

Guadalupe

Hale

Harris

Hays

Hemphill

Henderson

Hidalgo

Hockley

Hood

Jack

Jefferson

Jim Wells

Johnson

Jones

Kaufman

Kerr

Kleberg

Lamb

Liberty

Limestone

Lubbock

Matagorda

McLennan

Midland

Montague

Montgomery

Nacogdoches

Navarro

Nolan

Nueces

Palo Pinto

Panola

Parker

Potter

Rockwall

Rusk

Smith

Somervell

Starr

Stephens

Tarrant

Taylor

Terry

Titus

Tom Green

Travis

Tyler

Val Verde

Victoria

Walker

Webb

Wharton

Wichita

Wilbarger

Williamson

Wilson

Young

5. How many years have you been practicing medicine?

0-1

2-3

4-5

6-7

8-9

10-11

12-13

14-15

16-17

18-19

20 or more

6. In what type of community do you see patients? (Select all that apply)

Large city

Suburb, near a large city

Small city or town

Rural area

7. On average, how many patients do you treat in your practice per month?

1-499

500-1,999

2,000+

NTD attitude/knowledge questions

8. In your practice, do you evaluate patients with reportable diseases? A reportable disease is defined in Texas as those considered to be of great public health importance.

Yes

No

9. During your time in practice, how many NTDs (defined by the World Health Organization as a diverse group of conditions caused by a variety of pathogens and associated with devastating health, social, and economic consequences) have you diagnosed?

0

1

2

3

4

5

6

7

8

9

10

11

12

13

14

15

16

17

18

19

20 or more (if more than 20, please specify a number)

 9a1. If any number greater than 0: which of the following diseases have you diagnosed? (Select all that apply)

Buruli ulcer (*Mycobacterium ulcerans* infection)

Chagas disease (*American trypanosomiasis*)

Chikungunya

Dengue fever

Dracunculiasis (guinea-worm disease)

Echinococcosis

Foodborne trematodiases (Clonorchiasis, Opisthorchiasis, Fascioliasis, Paragonimiasis)

Human African trypanosomiasis (sleeping sickness)

Leishmaniasis

Leprosy

Lymphatic filariasis

Mycetoma

Chromoblastomycosis and other deep mycoses

Noma

Onchocerciasis

Rabies

Scabies and other ectoparasitoses

Schistosomiasis

Soil-transmitted helminthiases (including the round worm, *Ascaris lumbricoides*; the whipworm, *Trichuris trichiura* and hookworms, *Necator americanus* and *Ancylostoma duodenale*)

Snakebite envenoming

Taeniasis/cysticercosis (via *Taenia solium*, *Taenia saginata*, and *Taenia asiatica*)

Trachoma (*Chlamydia trachomatis* infection of the eye)

West Nile virus

Yaws (Treponema infections)

Zika virus

Other (please specify): ______

9a2: If any number greater than 0: what is your follow-up protocol? (Select all that apply)

Diagnostic testing

Supportive care

Report to the Centers for disease control and prevention (CDC)

Report to the Department of health and human services (DSHS)

Report to the Food and Drug Administration (FDA)

Report to the state of Texas

Referral to a specialist

Consult with a specialist

Hospitalization

No follow-up is done

Other (please specify): ______

9b. If ZERO: If you needed to report an NTD case, who would you contact?

Hospital infection control

Local health department

State health department

Centers for disease control and prevention (CDC)

Food and Drug Administration (FDA)

Department of health and human services (DSHS)

No one. Reporting should not be required

10. What symptom(s) is/are most indicative of an NTD based on your experience? (Select all that apply)

Fever

Diarrhea

Vomiting

Body aches

Rash

Bleeding

Headaches

Neck stiffness

Pain behind the eyes

Excessive sleepiness

Seizures

Loss of consciousness

Other (please specify): ______

11. In your opinion, which diagnostic testing is most appropriate for the following NTDs?

**Table 5:** NTD attitude/knowledge question 11 NTD: neglected tropical disease; ELISA: enzyme-linked immunosorbent assay; PCR: polymerase chain reaction; IFA: indirect fluorescent antibody

NTD	Clinical approach	Microscopy	ELISA	IFA	Plaque reduction neutralization test	Reverse transcription-polymerase chain reaction	Combination microscopy + PCR	Combination ELISA/IFA + PCR
Chagas disease (American trypanosomiasis)	-	-	-	-	-	-	-	-
Chikungunya	-	-	-	-	-	-	-	-
Dengue	-	-	-	-	-	-	-	-
West Nile virus	-	-	-	-	-	-	-	-
Zika virus	-	-	-	-	-	-	-	-

12. How important is it for physicians to inform public health authorities of reportable diseases?

Not at all important

Slightly important

Moderately important

Very important

Extremely important

13. Before taking this survey, were you aware of Texas law that states that “[h]ealth care providers, hospitals, laboratories, schools, and others are required to report to Texas Department of State Health Services (DSHS) patients who are suspected of having a notifiable condition.” (Health & Safety Codes Chapter 81, 84, and 87; Chapter 97, Title 25, Texas Administrative Code).

Yes

No

14. Before taking this survey, were you aware of the reportable disease list for contacting public health authorities about a reportable disease set forth by the Department of Health Commissioner?

Yes

No

15. Before taking this survey, were you aware of the required timeline for contacting public health authorities about reportable disease set forth by the Department of Health Commissioner?

Yes

No

16. Do you receive communication from the Texas Department of Health (either electronic or standard mail) regarding changes in Texas’ reportable disease list?

Yes

No

17. In your opinion, when is the best time to contact public health authorities regarding a patient with a reportable disease?

Upon suspicion of the diagnosis

When the patient clearly meets clinical criteria

When the disease is laboratory confirmed

When the patient has begun treatment

When the patient has completed treatment and responded

Never. Reporting should not be required

Other (please specify): ______

18. Deficits in which of the following knowledge areas may most contribute to incomplete reporting?

Requirement that providers report

Which diseases are reportable

Requirements for the timeliness of reporting

Mechanism for submitting a report to public health

Proper suspicion of the diagnosis

None of these

19. Deficits in which of the following knowledge areas may most contribute to delays in reporting?

Requirement that providers report

Which diseases are reportable

Requirements for the timeliness of reporting

Mechanism for submitting a report to public health

Proper suspicion of the diagnosis

None of these

20. In your opinion, who is best suited to contact public health authorities about a patient with a reportable disease?

Healthcare provider

Laboratory technician

Laboratory manager/supervisor

Infection control personnel

Automated (via electronic medical record)

No one. Reporting should not be required

Other (please specify): ______

21. What method of reporting is most convenient for you?

Secure email

Secure web-based form

Telephone

Secure fax

Mail

Other (please specify): ______

22. Reportable diseases have variable time frames for reporting. If you had a reportable disease to submit, how would you determine the required time frame to report?

Discuss with colleague

Discuss with the hospital infection control

Call the local or state health department

Access the local or state department of health website

Review posted reportable disease requirements

I am unsure of where to find the information

Other (please specify): ______

23. What is your primary concern regarding the notification of a reportable disease to public health authorities?

Concerns about violating patient privacy

Not worthwhile if the disease is not highly contagious/fatal

Reporting procedures are burdensome

Likely that someone else will report

Unsure which diseases are required to report

There is no concern

Other (please specify): ______
